# Pilot test and validation of the Peak Day method of prospective determination of ovulation against a handheld urine hormone monitor

**DOI:** 10.1186/1472-6874-14-4

**Published:** 2014-01-08

**Authors:** Christina A Porucznik, Kyley J Cox, Karen C Schliep, Joseph B Stanford

**Affiliations:** 1 Division of Public Health, Department of Family and Preventive Medicine, University of Utah School of Medicine, Salt Lake City, Utah, USA; 2 Division of Epidemiology, Statistics, and Prevention Research, Eunice Kennedy Shriver National Institute of Child Health and Human Development, National Institutes of Health, Rockville, Maryland, USA

**Keywords:** Environmental exposure, Epidemiology, Ovulation, Fertilization, Validation studies, Luteinizing hormone, Biomonitoring

## Abstract

**Background:**

Transient exposures may influence fertility and early embryonic development. To assess the time of conception *in vivo* and conduct concurrent biomonitoring, ovulation must be identified prospectively. We report on the development and validation of a simple, prospective method, the Peak Day method, to determine likely day of ovulation based upon daily observations of cervical fluid.

**Methods:**

We recruited 98 women to learn the Peak Day method from a brochure, 26 of whom concurrently used the method with blinded daily urine hormone monitoring (estrone glucuronide and luteinizing hormone). All women were instructed to complete an exposure questionnaire immediately upon identifying ovulation. Briefly, the exposure questionnaire captured time-varying and transient exposures such as medication use, water consumption, and amount of sleep. We assessed timely completion of the exposure questionnaire, agreement of women’s estimated day of ovulation (EDO) and the EDO by expert review, and agreement between the EDO by expert review and by blinded urine monitoring.

**Results:**

Of 147 cycles evaluated, women selected an EDO in 130 (88%) and subsequently completed the periovulatory exposure questionnaire in 122 (94%) cycles. Of the 26 cycles evaluated with blinded hormonal monitoring, the Peak Day “best quality” algorithm, based upon cervical fluid, identified ovulation ± 3 days of the urine monitor in 24 cycles (92%).

**Conclusions:**

With simple written instructions, women can identify an estimated day of ovulation and perform periovulatory exposure assessment. The Peak Day method is highly cost-effective and could be applied by researchers to target periconceptional or very early developmental stage exposure assessment.

## Background

In recent years, the Barker hypothesis initiated a revolution in our understanding of human development by proposing that events leading up to birth are associated with outcomes in adult life [1]. Evidence has accumulated for the effects of intrauterine environmental exposures on early development and adult health in both animals and humans, and now the developmental origins of non-communicable disease is a dynamic research area [2,3]. Environmental contamination by endocrine disrupting chemicals or mutagens during the fetal period affects virtually all organ systems in fetal development and throughout subsequent life [3-5]. The mechanism for such action may be that environmental exposures affect cellular stress, hormone regulation, or metabolic pathways leading to epigenetic changes in the organism [3]. Results from assisted reproduction techniques suggest that the environment to which the embryo is submitted affects genetic expression in ways that alter the phenotype of the organism throughout its subsequent development [5,6].

Limitations of exposure measurement methods hamper our ability to understand the effects of periconceptional environmental exposures. Exposures that are transient may have critical influences on fertility and the sensitive period of early embryonic development. Therefore, initial monitoring of key exposures should occur at or near the time of conception [7,8]. In order to assess the time of conception *in vivo*, the time of ovulation must be identified prospectively. Prospectively determining ovulation dates can identify precise time intervals between ovulation, conception, implantation, and subsequent development and would allow for targeted exposure assessment during the relevant developmental windows, such as fertilization and implantation [8-10].

A number of candidate biomarkers of ovulation exist, including hormonal biomarkers, menstrual cycle length, and symptom biomarkers [11,12]. Home ovulation detection kits based on urinary luteinizing hormone (LH) are reliable (ovulation by ultrasound occurring within 3 days of the urine LH surge in 97% of cycles); however, their daily use would become costly if used in large, population-based cohort studies [12-15]. A handheld computerized device based on urinary estrogen metabolites and LH has also been shown to be reliable in relation to biologic gold standards and to be effective in field studies [13,16-19]. However, its use may also be limited by cost considerations (currently up to $200 per monitor and > =$1 per day for test strips) [14,20].

Another frequently used attribute is cycle length, or fixed formulas for counting days of ovulation. The major limitation of this method is that there is wide variability in the length of the follicular (preovulatory) phase, as well as substantial variability in the length of the luteal (postovulatory) phase of the menstrual cycle [21-24]. Further, some common exposures, such as recent oral contraceptive use, systematically delay ovulation [25,26]. While adjustments can be made in calendar calculations for the purposes of estimating pregnancy rates, calendar-based approaches are not precise enough to assess periconceptional exposure. Using a calendar-based approach for the timing of ovulation will frequently be inaccurate by a week or more [21,22,24].

An alternative approach for identifying ovulation is the use of symptom biomarkers. Based on the available evidence, it has been proposed that using symptom biomarkers for ovulation may prove as accurate and effective as using hormonal biomarkers, at a much more cost effective ratio for large population-based studies [14,27]. Basal body temperature (BBT) rises soon after ovulation and is well correlated with the estimated day of ovulation (EDO) based on gold standard markers (including follicular ultrasound and serum hormonal measures), but cannot prospectively identify when ovulation is imminent [28]. Cervical fluid secretion increases greatly in quantity and changes in quality in the days preceding ovulation, resulting in characteristic changes that women can observe, changes that are also well correlated with gold standards for the EDO [28-30]. Because cervical fluid changes are necessary for sperm survival, they also accurately identify both the beginning and end of the fecund window (commonly called the fertile window), or the days when conception is most likely to occur [31,32].

Women can be taught to monitor cervical fluid and/or basal body temperature biomarkers for ovulation, and they can monitor them reliably and interpret them accurately as has been demonstrated in numerous field studies, including international settings [33,34]. However, because previous work has focused on monitoring for family planning purposes, it has involved relatively intensive instruction [35-37] (i.e., four or more personal sessions with an instructor), which would limit applicability for epidemiologic assessment in large populations. We report on the pilot testing and validation of a basic written brochure (printed or online) for a streamlined, prospective method to identify the fecund window and likely day of ovulation based upon self-observation of changes in cervical fluid, with an option for additional use of basal body temperature. Our motivation is to develop a tool for environmental epidemiologists wishing to link environmental exposure assessment with the periconceptional period or subsequent developmental windows more precisely than is possible with calendar-based tools. Such precision may not be relevant for exposures that are continuous, but the timing of exposure assessment may affect greatly the measured values for time-varying exposures. For example, we are measuring bisphenol A, an endocrine disruptor, in daily first morning urine samples in an ongoing cohort and have found daily within-person variation as much as twenty fold within a single menstrual cycle.

## Methods

### Study population

Ninety-eight women, ages 18–44, who were not currently using hormonal contraception were recruited for the Peak Day pilot or validation studies during 2009–2011. Women were not required to be sexually active or trying to conceive to participate. Women were recruited through friend referrals, flyers, websites and local community groups. In addition, based upon data within the Utah Population Database, letters were mailed to women identified as married for at least two years who had not yet experienced a live birth. Women in the pilot study could live anywhere but women who participated in the validation sub-study were restricted to the Salt Lake City, Utah area. The University of Utah Institutional Review Board and the Utah Resource for Genetic Epidemiologic Research approved the study and written informed consent was obtained from all participants prior to enrollment. After consenting, women completed an online enrollment questionnaire. In addition to demographic information and reproductive history, participants also recorded their current pregnancy intention (scale 0–10, 0 = trying very hard to avoid pregnancy and 10 = trying very hard to achieve pregnancy), typical level of physical activity, smoking status, alcohol consumption, weekly fruit and vegetable intake, and previous knowledge or use of fertility awareness-based methods. A follow-up phone call or email was conducted in attempt to complete pertinent missing enrollment questionnaire data.

### The Peak Day method

In an iterative process involving experts in the fields of natural family planning and fertility awareness, we have created a streamlined, simplified approach to fertility awareness instruction to identify the EDO, called the Peak Day method. Participants in the Peak Day studies received three sample fertility charts accompanied by a three-page brochure (printed or online at http://medicine.utah.edu/dfpm/OCRH/peakday/Brochure.pdf) that explained how to observe, record, and interpret characteristics of the cervical fluid in order to identify the likely day of ovulation. In the brochure, participants were instructed to make external vulvar observations for the presence of cervical fluid during routine use of the bathroom and to record each day that slippery, stretchy, and/or clear fluid was present. They were informed that their EDO is the last day during a menstrual cycle when they observed fluid with at least one of these fertile qualities, i.e., the “last fertile sign” algorithm. In most women, cervical fluid diminishes quickly following ovulation in response to the increased levels of progesterone with an accompanying rise (~0.3°F) in temperature. Thus, the EDO can be determined 1–2 days after it occurs and the fecund window with the days leading up to ovulation can be identified prospectively [11,14]. Information about seminal fluid and arousal fluid, which can mimic cervical fluid and are critical for pregnancy-avoidance fertility awareness-based methods, were not included in the participant brochure given that the Peak Day approach is simplified and not all participants were sexually active or trying to conceive.

Instruction about measuring, recording, and interpreting basal body temperature was included in the Peak Day educational brochure as an optional additional biomarker that could be used to confirm the occurrence of ovulation. Women could return their paper charts to study staff via mail or enter their daily observations securely online. The study coordinator was available by phone and email to answer questions and made certain that all participants understood that Peak Day method should not be used to avoid pregnancy.

### Pilot study

**Figure 1:**
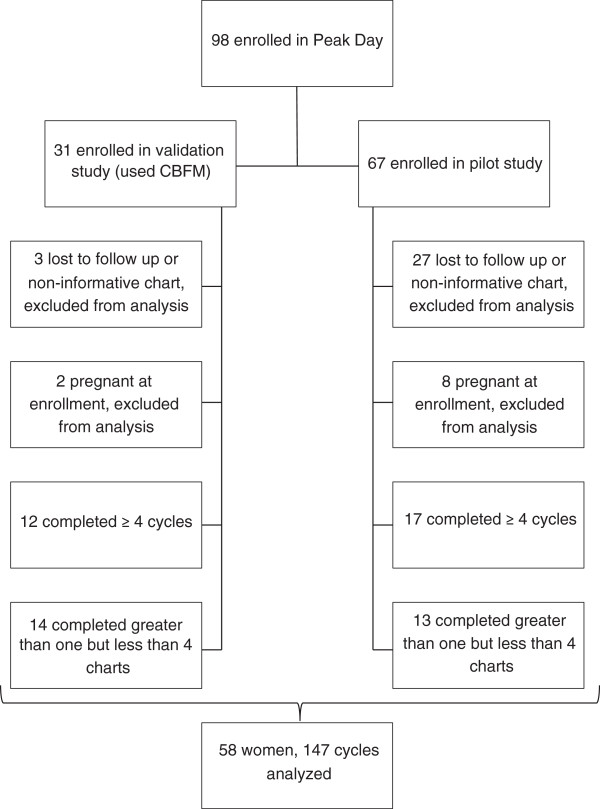
Participant flowchart.

Of the 98 women recruited for the Peak Day studies, 67 women enrolled in the pilot study alone, to evaluate the acceptability and usability of the method (Figure 1). Women reviewed the educational materials and then recorded their observations of cervical fluid (and basal body temperature, if desired) and selected Peak Days for up to six menstrual cycles or until they became pregnant. We asked the women to complete a short exposure questionnaire once she determined her EDO, typically at EDO + 1 day. The purpose of this was to demonstrate that women could both identify their EDO and subsequently perform an exposure assessment task that recorded exposures that occurred on the day of ovulation.

### Validation sub-study

An additional 31 women enrolled in the validation sub-study in addition to the pilot, agreeing to daily test their first morning urine using a blinded research version of the ClearBlue® Easy Fertility Monitor (CBFM) (Inverness Medical, Waltham MA, USA). These women also performed daily recording of fertility biomarkers according to the Peak Day method, and completed periovulatory exposure questionnaires for two cycles or until becoming pregnant. The clinical version of the CBFM was developed to assist women in becoming pregnant by identifying their fertile window through measurements of both estrone-3-glucuronide (E3G) and LH in urine. High fertility indicates urinary E3G (typically between 20 and 30 ng/mL) that correlates with the later follicular phase estrogen rise [13]. The CBFM continues to register a high fertility reading until a threshold of urinary LH is detected, typically >30 IU/I, indicating peak fertility [13]. Validation research of the CBFM has found that the day of ovulation occurs one day after the CBFM urine LH surge (or the first day that peak fertility is indicated) for the majority of cycles [13]. The research version of the CBFM for the Peak Day validation sub-study was blinded so that participants could not receive information regarding their fertility status, but rather, daily results were stored on a data card, which we downloaded at the end of each cycle.

### Fertility chart expert review

During the course of the pilot study, we observed a number of cycles with fertile-type observations in the few days immediately prior to menses. Since premenstrual fluid can sometimes mimic fertile-type cervical fluid [38], several participants incorrectly chose Peak Days according to the “last fertile sign” algorithm based on these premenstrual observations. Given that the Peak Day method is streamlined and self-taught, it became apparent that the instructions did not adequately clarify the distinction between true fertile-quality cervical fluid and premenstrual fluid. To address this, expert reviewers were used to select the Peak Day to simulate what a woman would have likely picked had she been given more precise instructions about choosing between true cervical fluid and premenstrual fluid.

We decided to test an additional algorithm, one that might eliminate the need for the distinction between premenstrual fluid and true cervical fluid because one is asked to observe the quality of cervical fluid rather than choosing the last day that fluid is present. The “best quality” algorithm identifies the last day during a menstrual cycle when a woman observed the *most* number of the three fertile-type cervical fluid characteristics, i.e., slippery, stretchy, and/or clear. A subset of participants was instructed to use this “best quality” algorithm for comparison to the “last fertile sign” algorithm.

For all cycles in the study, two independent, blinded expert reviewers noted the cycle day the woman identified as her Peak Day and whether she completed the exposure questionnaire for her self-identified Peak Day. Additionally the expert reviewers made their own assessment of a Peak Day based on the “last fertile sign” algorithm and on the “best quality” algorithm. Expert reviewers excluded fertile-type cervical fluid observations seven days prior to menses (premenstrual fluid) for both algorithms.

Where the two expert reviewers disagreed, an adjudicating assessment was made by a third expert reviewer. This adjudicating assessment was needed in 12 (11%) cycles for the “last fertile sign” algorithm and in four (4%) cycles for the “best quality” algorithm. Both the “last fertile sign” and “best quality” algorithms were evaluated in order to determine the most valid self-identified biomarker of ovulation for peri-ovulational data collection using Peak Day (test method) as compared to the first day after the urine LH surge was identified by CBFM (reference method).

### Statistical analyses

Descriptive statistics were calculated to summarize participant demographic and charting characteristics (Table 1), as well as completion rates for determining EDO and questionnaire exposure assessments. To assess women’s ability to learn and correctly use the Peak Day method for identifying their EDO (pilot study), we calculated percent agreement between women’s identified “last fertile sign” EDO (method learned via instructional brochure) and expert review of “last fertile sign” EDO as well as “best quality” EDO (method learned by a subset of participants via instructional brochure) and expert review of “best quality” EDO (Table 2). We conducted two sensitivity analyses: 1) we excluded women who had previously used a fertility awareness-based method to clarify how well women can learn to identify their EDO exclusively using the Peak Day method; and 2) we assessed agreement after excluding cycles with basal body temperature recordings to assess how well women can learn to identify their EDO using cervical fluid observations alone.

**Table 1:** Selected characteristics of study participants

	**Total mean ± SD, or n(%) n = 58**	**Pilot mean ± SD, or n(%) n = 32**	**Validation mean ± SD, or n(%) n = 26**
Age*	28.1 ± 5.8	29.1 ± 4.5	26.8 ± 7.2
**BMI**^**†**^	23.8 ± 5.5	23.7 ± 4.5	23.9 ± 6.9
<18.5	5 (8.6)	2 (6.3)	3 (11.5)
18.5-24.9	34 (58.6)	20 (62.5)	14 (53.9)
25.0-29.9	6 (10.3)	5 (15.6)	1 (3.8)
≥30	8 (13.8)	4 (12.5)	4 (15.4)
Missing	5 (8.6)	1 (3.1)	4 (15.4)
**Physical activity**^**‡**^	3.2 ± 1.4	3.1 ± 1.3	3.4 ± 1.5
**Age at menarche**	12.5 ± 1.5	12.7 ± 1.4	12.4 ± 1.8
**Gravidity**	0.8 ± 1.2	1.0 ± 1.5	0.4 ± 0.6
**Parity**	0.8 ± 1.1	1.0 ± 1.3	0.5 ± 0.5
**Race**			
Non-Hispanic White	50 (86.2)	29 (90.6)	21 (80.8)
Hispanic White	2 (3.5)	1 (3.1)	1 (3.8)
Other/Multiracial^£^	1 (1.7)	0	1 (3.8)
Missing	5 (8.6)	2 (6.2)	3 (11.5)
**Employment**			
Employed for wages	27 (46.5)	14 (43.8)	13 (50.0)
Self-employed	4 (6.9)	3 (9.4)	1 (3.8)
Homemaker	15 (25.9)	9 (28.1)	6 (23.1)
Student	6 (10.3)	4 (12.5)	2 (7.7)
Unemployed/other	3 (5.2)	1 (3.1)	2 (7.7)
Missing	3 (5.2)	1 (3.1)	2 (7.7)
**Education**			
High School/GED or < High School	9 (15.5)	6 (18.7)	3 (11.5)
College graduate	45 (77.6)	24 (75.0)	21 (80.8)
Missing	4 (6.9)	2 (6.3)	2 (7.7)

^*^Age in years.

^†^Body Mass Index: weight (kg)/height (m)2.

^‡^Number of days per week.

^£^Includes Asian, Black/African American, Pacific Islander, American Indian/Alaskan Native.

**Table 2:** Agreement between woman-selected estimated date of ovulation and blinded expert review according to the peak day method of fertility tracking

		** Scenario**		
		**Woman selected EDO**	**Woman selected EDO excluding women who previously used a fertility awareness-based method**	**Woman selected EDO excluding cycles for which basal body temperature was recorded**
Window in relation to EDO as assessed by expert review using the “last fertile sign” algorithm^1^	Cycles	n = 110	n = 82	n = 67
	Exact day n(%)	84 (76)	64 (78)	52 (78)
	± 1 day n(%)	90 (82)	68 (83)	56 (84)
	± 2 days n(%)	96 (87)	72 (88)	59 (88)
	± 3 days n(%)	102 (93)	76 (93)	61 (91)
Window in relation to EDO as assessed by expert review using the “best quality” algorithm^2^	Cycles	n = 20	n = 10	n = 9
	Exact day n(%)	18 (90)	8 (80)	7 (78)
	± 1 day n(%)	19 (95)	9 (90)	8 (89)
	± 2 days n(%)	20 (100)	10 (100)	9 (100)
	± 3 days n(%)	20 (100)	10 (100)	9 (100)

*Abbreviations:* EDO (estimated day of ovulation).

^1^Last day during a menstrual cycle when a woman observed any of the three fertile-type cervical fluid characteristics.

^2^Last day during a menstrual cycle when a woman observed the most number of fertile-type cervical fluid characteristics.

To determine the validity of the two Peak Day algorithms (validation sub-study) compared to urine hormone monitoring, we calculated percent agreement for EDO based on expert review of the “last fertile sign” and “best quality” Peak Day algorithms, and the CBFM for the same day, and within ≤1, ≤2, and ≤3 days. We additionally assessed agreement for expert review of the “last fertile sign” and “best quality” after excluding fertile-type cervical fluid observations seven days prior to menses.

## Results

Of the total consented population, 10 women became pregnant before beginning to chart and 14 women had one or more uninformative cycles (n = 30 cycles) due to medical issues (e.g., anovulation or continuous cervical fluid) or incomplete charts (n = 4 cycles), leaving 58 women contributing a total of 147 cycles for analysis. The study population was predominately white, non-Hispanic (86%) and educated (77% completing college or above) with a mean age of 28.1 years (standard deviation [SD] =5.8), and Body Mass Index (BMI) of 23.8 kg/m^2^ (SD = 5.5) (Table 1). Forty-one percent of women had previously been pregnant, 36% previously had a live birth, and 87% had previously used hormonal contraception. A majority of women (72%) had heard of fertility awareness-based methods, but a minority (28%) had previously tried one or more fertility awareness-based methods: ovulation urine test kits (n = 9); cervical fluid with basal body temperature observation (n = 8); calendar-based method (n = 3); and cervical fluid observation alone (n = 2).

Women contributed a mean of 3.2 cycles (SD =1.9 cycles) to the study. Among the 147 cycles, women recorded cervical fluid observations alone in 61% and both cervical fluid observations and basal body temperature in 39%. The estimated date of ovulation (EDO) was marked in 88% of cycles and the Peak Day exposure questionnaire was completed in 94% of cycles for which an EDO was marked. The majority (40/58 = 69%) of women wanted to conceive (i.e., pregnancy intention score on study entry of ≥5 out of 10) and of those wishing to get pregnant, most conceived within 4 cycles (31/40 = 78%).

Among all cycles in which an EDO was recorded by women using the “last fertile sign” algorithm, (n = 110), participants selected the correct EDO in comparison to the expert-selected “last fertile sign” algorithm in 76% of cycles, ± 1 day in 82% of cycles, ± 2 days in 87% of cycles, and ± 3 days in 93% of cycles. Excluding women who had previously used a fertility awareness-based method slightly improved agreement. Excluding cycles with basal body temperature observations slightly decreased agreement between participant-selected EDO and expert selected EDO. Agreement was higher when participants used the “best quality” algorithm (n = 20). Participants selected the correct EDO in comparison to expert-selected exactly in 90% of cycles, ± 1 day in 95% of cycles, and ± 2 days in 100% of cycles. When women who had previously used a fertility-based method were excluded, agreement decreased to 80% agreement for an exact match and to 90% ± 1 day. There was no change in agreement for ± 2 and ± 3 days. Agreement on the exact day and ± 1 day dropped slightly more when cycles with basal body temperature observations were excluded, but again, remained unchanged for ± 2 and ± 3 days (Table 2).

In the validation sub-study (n = 26), the “last fertile sign” algorithm as identified by the expert reviewers selected the same day as the CBFM in 19% of cycles, ± 1 day of the monitor in 54% of cycles, and ± 2 and ± 3 days of the monitor in 77% of cycles. In comparison, the “best quality” algorithm had higher sensitivity: it identified the same day as the monitor in 19% of cycles, ± 1 day of the monitor in 65% of cycles, ± 2 days of the monitor in 88% of cycles, and ± 3 days of the monitor in 92% of cycles (Table 3). Excluding the seven days prior to menses improved agreement for “last fertile sign” algorithm, but made no difference in agreement with “best quality” algorithm since the premenstrual cervical fluid was present but did not have the same degree of fertile characteristics as observed around ovulation.

**Table 3:** Sensitivity of peak day method for determining estimated day of ovulation compared to Clearblue® Easy fertility monitor in 26 cycles

	**Exact day**	**≤1 day**	**≤ 2 days**	**≤ 3 days**	**>3 days**
	**n (%)**	**n (%)**	**n (%)**	**n (%)**	
“Last fertile sign^1^” algorithm including seven days prior to menses	4 (15)	13 (50)	18 (69)	18 (69)	8 (31)
“Last fertile sign^1^” algorithm excluding seven days prior to menses	5 (19)	14 (54)	20 (77)	20 (77)	6 (23)
“Best quality^2^” algorithm including seven days prior to menses	5 (19)	17 (65)	23 (88)	24 (92)	2 (8)
“Best quality^2^” algorithm excluding seven days prior to menses	5 (19)	17 (65)	23 (88)	24 (92)	2 (8)

^1^Last day during a menstrual cycle when a woman observed any of the three fertile-type cervical fluid characteristics.

^2^Last day during a menstrual cycle when a woman observed the most number of fertile-type cervical fluid characteristics.

## Discussion

Our findings demonstrate that women are both able to identify their EDO following self-instruction in fertility biomarkers (primarily cervical fluid) and willing to complete an exposure assessment task when they identify their EDO. Our results suggest that the likely day of ovulation +/− 3 days can be reliably determined using the Peak Day “best quality” algorithm, with no apparent benefit (or detriment) from additionally using BBT observations. The “best quality” had higher sensitivity to detect the EDO compared to the “last fertile sign” algorithm. The novel contributions of this study are the viability of streamlined, self-instruction to identify the EDO, and the higher sensitivity to identify the timing of ovulation when using the “best quality” algorithm, as compared to our original “last fertile sign” algorithm. For a large, population-based cohort study of couples trying to conceive, neither in-person instruction nor daily hormone monitoring are logistically or economically feasible, and the Peak Day method represents a cost-efficient alternative for determining ovulation for the purposes of periovulatory/periconceptional exposure assessment. Additionally, researchers could use the Peak Day EDO information to establish dates for blood draws timed to developmental windows during pregnancy.

Although some women chose to use BBT as an additional sign in a large proportion of cycles (39%), we did not find any evidence that use of BBT improved accuracy. However, we cannot exclude the possibility that women self-selected somewhat more difficult cycles for BBT use where BBT influenced the observations and interpretations to improve accuracy. The slightly increased agreement when excluding women who have previously used other fertility awareness-based methods suggests that women with previous training may have a more difficult time exclusively using the Peak Day method for identifying EDO and might benefit from special instruction on how not to confuse methods.

Several other approaches to observing cervical fluid exist but none are self-taught without assistance of a trained teacher of natural family planning or nurse, and teach women to identify an EDO. One simplified instructional approach and algorithm for observing cervical fluid (TwoDay Method) has been developed and validated for family planning purposes; however, it does not teach women to identify an estimated day of ovulation [39]. The Creighton Model Fertility Care System teaches women to observe cervical fluid using the same three characteristics as the Peak Day method but it requires personal instruction with a trained teacher over a period of several months [35-37]. The Marquette University Natural Family Planning (NFP) website [nfp.marquette.edu] allows users to learn about NFP methods online and chart there with primarily self-taught methods, but the website is actively managed by nurses and physicians that interact with participants [40]. Unlike the Creighton Model Fertility Care System and the Marquette University NFP website, the Peak Day method is not designed for family planning, nor for medical applications such as evaluation and management of infertility [38].

A recently published study similarly compared participant perceived peak days of fertility to CBFM identified peak days, but without use of cervical mucus monitoring [41]. In that study, only 56% of women estimated the day of ovulation within ±2 days of the CBFM identified peak day [41], much less than the 88% agreement seen with the “best quality” algorithm when compared to CBFM. This suggests that cervical mucus monitoring is an accurate and efficient way for women to prospectively identify ovulation without need for an external hormone monitoring device.

The Peak Day method identifies a much more precise window of the periconceptional period than that used in most previous studies of periconceptional exposures. Prior studies have used various definitions of the periconceptional period ranging from the month of conception [42,43], to a 2–3 month window including conception [44-47], to a six month window including conception [48,49], to the three months prior to and during the pregnancy [50,51], and the year before the pregnancy [52]. In addition, many previous studies of periconceptional exposures and pregnancy outcomes have relied on maternal recall of past exposures [42,43,45,48-52]. Such means of timing exposure assessment may be adequate for exposures that are habitual or easy to recall but would be ineffective for exposures that are transient or require analysis of biospecimens. Further, environmental exposures that affect probability of conception or time to pregnancy (TTP) can only be studied in prospective cohorts because of the bias inherent in recruiting pregnant women into studies. Application of the Peak Day method in a cohort would allow for concurrent measurement of exposure via diaries, biomonitoring, or short-term recall.

Retrospective exposure measurement hampers our ability to understand the effects of periconceptional environmental exposures. The Peak Day method could be applied in prospective studies of fecundability to address these limitations. We are presently using Peak Day (“best quality” algorithm) to time biospecimen collection and questionnaire-based exposure assessment for bisphenol-A (BPA) in a prospective cohort of couples trying to conceive. Because of the importance of the male partner in explaining TTP variation, we are using the Peak Day method to assess both the female and male periconceptional exposure to BPA [53]. It is important to emphasize, however, that the Peak Day method could be applied in the context of targeting exposure assessment for any time-varying exposure.

A minority of women in this study had prior experience with self-observation of fertility signs, but there was good acceptance of the method as indicated by completion of the protocol. We are using the same method in our current preconception cohort, and it is well-accepted by this population trying to conceive. We are unable to speculate how acceptable the method would be to a general population of women.

Further studies are needed to assess how well the Peak Day method can be learned and used by less educated women. Previous studies have shown that women of low educational status can learn and reliably use other cervical-fluid fertility awareness methods for family planning,[39,54] so we are optimistic for the potential of the Peak Day method in such populations, possibly with some adaptation. We had a limited number of cycles to assess validity of the Peak Day algorithms compared to the CBFM, and a limited number to assess whether concurrent basal body temperature observations or previous use of other fertility-awareness based methods affect validity. Further research should confirm that women can apply the “best quality” algorithm to identify their EDO directly and more systematically address the impact of additionally recording their BBT (e.g., possibly by a randomized assignment to additionally using BBT). Finally, while the Peak Day method is appropriate for women of normal fertility, it is not appropriate for women with some menstrual cycle disorders, including oligomenorrhea or continuous cervical fluid.

## Conclusions

In summary, the Peak Day method is a novel and simple method for timing assessments for exposures relevant to fertility, pregnancy, and perinatal outcomes that would be difficult or impossible to assess retrospectively with precision. In 92% of cycles in this pilot study, the likely day of ovulation ± 3 days was reliably determined using the Peak Day “best quality” algorithm. Women completed the exposure questionnaire as instructed in 94% of cycles for which an EDO was identified. The Peak Day method is promising for studies of environmental exposures in very early pregnancy, where prospective determination of ovulation/conception is essential and the large population required to detect small effects may make daily urine hormone monitoring cost-prohibitive.

## Abbreviations

EDO: Estimated day of ovulation; LH: Luteinizing hormone; CBFM: Clearblue® easy fertility monitor; E3G: Estrone-3-glucuronide; BBT: Basal body temperature; NFP: Natural family planning; TTP: Time to pregnancy; BPA: Bisphenol-A.

## Competing interests

Separate from this study, author Joseph Stanford served as a paid consultant for Swiss Precision Diagnostics in regards to designing a clinical trial and interpreting results of a clinical trial for a urine pregnancy test. The other authors declare that they have no competing interests.

## Authors' contributions

CP participated in the study design, supervised data analysis and assisted in drafting and revising the manuscript; KC carried out the data collection, statistical analysis, and assisted in drafting and revising the manuscript; KS participated in study design, data collection, statistical analysis, and drafting and revising the manuscript, JS participated in study design and drafting and revising the manuscript. All authors read and approved the final manuscript.

## Pre-publication history

The pre-publication history for this paper can be accessed here:

http://www.biomedcentral.com/1472-6874/14/4/prepub
